# Translational Science by Public Biotechnology Companies in the IPO“Class of 2000”: The Impact of Technological Maturity

**DOI:** 10.1371/journal.pone.0082195

**Published:** 2013-12-16

**Authors:** Laura McNamee, Fred Ledley

**Affiliations:** Center for Integration of Science and Industry, Bentley University, Waltham, Massachusetts, United States of America; Merck & Co., United States of America

## Abstract

The biotechnology industry plays a central role in the translation of nascent biomedical science into both products that offer material health benefits and creating capital growth. This study examines the relationship between the maturity of technologies in a characteristic life cycle and value creation by biotechnology companies. We examined the core technology, product development pipelines, and capitalization for a cohort of biotechnology companies that completed an IPO in 2000. Each of these companies was well financed and had core technologies on the leading edge of biological science. We found that companies with the least mature technologies had significantly higher valuations at IPO, but failed to develop products based on these technologies over the ensuing decade, and created less capital growth than companies with more mature technologies at IPO. The observation that this cohort of recently public biotechnology companies was not effective in creating value from nascent science suggests the need for new, evidence-based business strategies for translational science.

## Introduction

The classical path for translational science distinguishes the roles of two different enterprises; a scientific enterprise focused on the basic research that generates new knowledge and technological capabilities, and a commercial enterprise that is responsible for translating these advances into products through applied research and development. In the case of new therapeutic products, the scientific enterprise primarily involves government-funded research that generates knowledge of disease pathogenesis, therapeutic strategies, potential targets for therapeutic interventions, new classes of therapeutic entities, and sometimes lead product candidates. These nascent scientific advances are classically transferred to a commercial enterprise, commonly a biotechnology company funded through capital markets, that is expected to pharmaceuticalize this science into finished products, conduct pre-clinical and clinical research, establish scalable production and quality control capabilities, achieve regulatory approval, and finally establish a marketing, sales, distribution, and service network required to make the product available to the public. The biotechnology industry, thus, has a dual mission of developing products from advances in basic sciences and generating capital growth to provide investors a positive return on their investments. Despite the enormous progress and promise of biomedical science, the biotechnology industry has largely failed to produce either a robust pipeline of new biopharmaceutical products[1], [2] or sustained economic returns.[3].

In this paper, we consider how the maturity of technologies through a quantifiable life cycle may contribute to the efficiency of translational science. Pisano has observed that many biotechnology companies are founded with very early-stage science that has not yet produced proof of principle or candidate products. He has suggested that such companies represent a “science based business,” in which value is created primarily by continued advances in scientific knowledge[3]. In contrast, other companies are founded with more mature science or technology that has already provided validated targets, lead candidates, or even previously marketed products.

To explore how the maturity of a company's technology impacted its dual mission of developing products and generating capital growth, we studied a cohort of 46 biotechnology companies that completed their IPO in 2000 and were focused on developing or improving therapeutic products. We asked how the maturity of each company's core technologies at the time of IPO impacted their ability to translate this science into therapeutic products and create capital growth in the first decade after their IPO.

The biotech “class of 2000” provides a useful experimental model in which to study the impact of technological maturity for several reasons. First, biotechnology IPOs are historically cyclic[4], and the 2000 IPO window was the last time that a large number of biotechnology companies completed IPOs prior to 2012–2013. Second, each of the 46 companies that completed an IPO in this window was a well-established corporate entity, with adequate capital resources following its IPO, as well as core technologies and market opportunities that attracted the interest of investment bankers, institutional investors, and analysts. Thus, this cohort excluded inadequately capitalized and organized start-up enterprises. Third, each of these companies in the “class of 2000” was subject to the same market conditions, investment and partnering trends, and regulatory environment in the decade after their IPO, thus enhancing the statistical power of this analysis. Finally, recognizing that the IPO environment of 2000 may not have been typical, the focus of this work was on product development and economic value creation in the ensuing decade, and this cohort of companies allowed us to examine performance in a context that was the most relevant to today's biotechnology industry.

**Figure 1:**
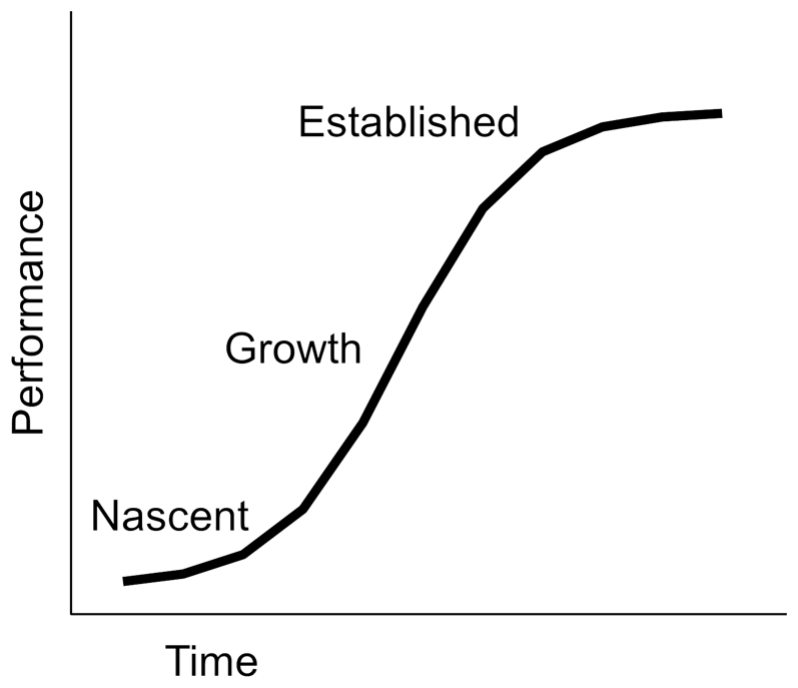
Technology life cycle follows an S-curve. *Established* Technology Companies (ETCs) utilize mature technologies that are approaching their limit. *Growth* Technology Companies (GTCs) utilize technologies that are in the rapid growth phase. *Nascent* Technology Companies (NTCs) utilize new technologies.

Technological innovation in many fields has been described as progressing through a characteristic Technology Life Cycle[5], [6]. This life cycle starts with a *Nascent* stage often characterized by salient technological advances, insights or invention, which leads to a period of exponential *Growth* in knowledge or technological capabilities. As the technology becomes *Established*, advances slow and limits are inevitably encountered, and the leading edge of research moves to new discoveries and technologies. The character of this technology life cycle is often described as an “S-curve,” and can be modeled as a logistic regression (Figure 1).[5], [6].

The S-curve is at the core of extensive theoretical work on technological innovation. For example, Foster observed that investments in *Nascent* technologies may be effective in advancing technological capabilities, but do not lead to products that can compete effectively with established technologies in existing markets. Conversely, investments in *Established* technologies are less likely to produce technological improvements, but are more likely to provide competitive products.[5] Christensen and Raynor have similarly shown how technological maturity impacts markets and competitive strategies. Their analysis suggests that *Established* technologies tend to sustain existing markets where they may improve or extend the features that customers already expect. In contrast, *Nascent* technologies often fail to meet the standards of existing markets and are more successful when introduced into unserved or low-end markets where customer expectations are different.[6] Finally, it has been observed that the development of *Nascent*, potentially disruptive technologies may require management strategies and structures that are very different from those that are effective in developing more mature technologies.[6], [7].

Most of the theoretical work on technology life cycles and innovation has emerged from fields where technological capabilities can be easily measured, such as computer and information technologies. In a previous paper, we examined the application of these theories to biotechnology. We used bibliometrics to examine the technology life cycles for three classes of novel therapeutics—gene therapy, monoclonal antibodies, and oligonucleotide therapeutics—and observed patterns of maturation similar to those in other fields. Importantly, for monoclonal antibodies, we observed an association between the stage of the technology life cycle and success in clinical development. Only one product candidate that entered clinical trials early in the technology life cycle led to approval, and this product was strategically withdrawn from the market in response to competitive advances. Only when monoclonal antibody technologies matured was there a pipeline of products entering clinical trials that led to successful products.[8].

In this work, we extend these observations to consider the impact of technological maturity through this characteristic life cycle on the ability of biotechnology companies to translate this science into products and capital growth. Examining a cohort of 46 companies that completed an IPO in 2000, we found that companies with nascent technologies at IPO had significantly higher valuations and provided venture capital investors with a higher step-up in valuation than companies with more established technologies. Over the ensuing decade from 2000–2010, however, these companies were unable to develop any products from these technologies, and they achieved significantly less product development and capital growth than companies with more established technologies. These results are analyzed in the context of innovation theories that highlight the importance of a fit between the position of a technology in its life cycle and the business model to commercialize that technology. These theories, developed in other technology-driven sectors, may be relevant to improving the efficiency of the translational science in biotechnology.

## Results

### The biotech class of 2000

Investment in biotechnology is historically cyclic and industry analysts have identified four windows for biotechnology IPOs since 1990.[4], [9] The shortest and most dramatic window was between October 1999 and September 2000, when 46 biotechnology companies focused on developing or improving therapeutic products completed IPOs. This window spans the zenith of the “dot com bubble” in March 2000 when NASDAQ and the NASDAQ Biotechnology Index (NBI) closed at all-time highs of 5132 and 1619 respectively.

This unique cohort of companies provided an ideal opportunity to study the impact of technological maturity on value creation in a controlled manner. Specifically, each of these companies was well established and subject to the same market conditions, investment and partnering trends, and regulatory environment (especially with regard to patent protection, drug approvals, and reimbursement), which enabled a rigorous statistical analysis focused on the effect of technology maturation. The 2000 IPO window also coincided with a period of rapid progress in genomics and the much-celebrated completion of the first human genome sequence, announced in June 2000. The cohort included 14 companies that were focused on applications of genomics, including six in pharmacogenomics.

Of the 46 companies studied, 44 were originally founded as venture-backed start-ups. The companies had collectively raised $2.8B in private capital investments prior to IPO (range $52M–$207M), and had a combined pre-money valuation of $15.4B (range $102M–$967M). All had a pre-money valuation greater than capital invested, with a median pre-money valuation six times the amount of prior investments (range 2–23 times). At IPO, these companies collectively raised $4.4B (range $35M–$244M) and had a collective post-money valuation of $19.8B (range $137M–$1,200M). No companies had positive earnings, and there was no correlation between the IPO valuation or capital raised at IPO and previous capital investments or revenues.

### Core technologies

Each company's core technology was identified from regulatory filings required for their IPO (S-1) and classified as *Established*, *Growing* or *Nascent* using the criteria described. The 16 companies with *Established* stage technologies (ETC) included companies developing monoclonal antibodies, managing late-stage product development, or reformulating drugs for new disease indications. The 12 companies with *Growing* stage technologies (GTC) were focused on rational drug design, combinatorial chemistry, high throughput screening, or gene therapy. The 18 companies with *Nascent* stage technologies (NTC) were focused on such fields as genomics, pharmacogenomics, proteomics, or directed evolution.

**Figure 2:**
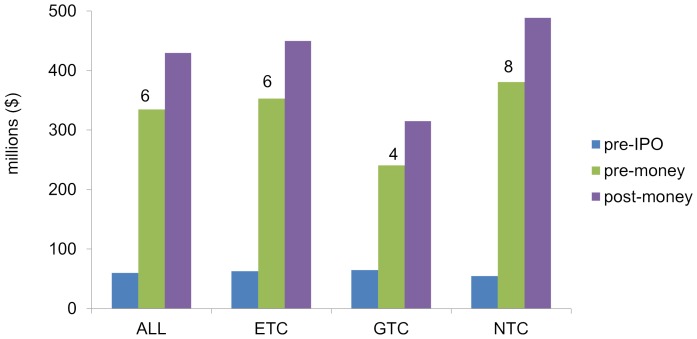
Capital investment and valuations for each company class at IPO. Average capital investment prior to IPO (blue bars); Average pre-money valuation at IPO (green bars); Average post-money valuation at IPO (purple bars). The number over each of the green bars represents average ratio of pre-money valuation at IPO to capital invested prior to IPO. The average pre-money valuation of NTCs was significantly higher than GTCs, or ETCs and GTCs combined. NTCs on average raised significantly more capital at IPO than GTCs and had significantly higher average post-money valuation than GTCs (p<0.05).

At the time of IPO in 2000, there was no statistically significant difference in the ages of companies in these three classes. NTCs had raised less capital prior to IPO than either GTCs or ETCs, though the difference was not significant. At IPO, the pre-money valuations of NTCs averaged eight times capital raised, which was significantly higher than GTCs or ETCs and GTCs combined. NTCs also raised significantly more capital at IPO than GTCs and had significantly higher post-money valuation than GTCs (Figure 2).

In S-1 filings, 22/46 companies described having candidate products in clinical development. A total of 36 candidate products were described. ETCs had more products in development than GTCs (Table 1). Only one had a product on the market, which had been acquired after regulatory approval. NTCs had no products in development. Candidate products included a variety of chemical entities such as small molecules, proteins, gene therapies, and cell therapies with clinical targets spanning infectious, inflammatory and cardiovascular diseases, as well as pain, cancer, and dementia. There was no correlation between pre-money or post-money valuations at IPO and the number of products in development, their most advanced clinical phase, Predicted Product Approvals (PPAs), or the Present Value of products in development.

**Table 1:** Products in clinical development at IPO.

	Phase 1	Phase 2	Phase 3	NDA	PPA	Approved
ETC	3	14	4	2	8	0
GTC	8	3	2	0	3	0
NTC	0	0	0	0	0	0

### Capital growth

At the end of 2010, ten years after the 2000 IPO window, 28/46 companies were still active, including 10/18 NTCs, 8/12 GTCs, and 10/16 ETCs. Of the others, 16 had been acquired and two had declared bankruptcy.

**Figure 3:**
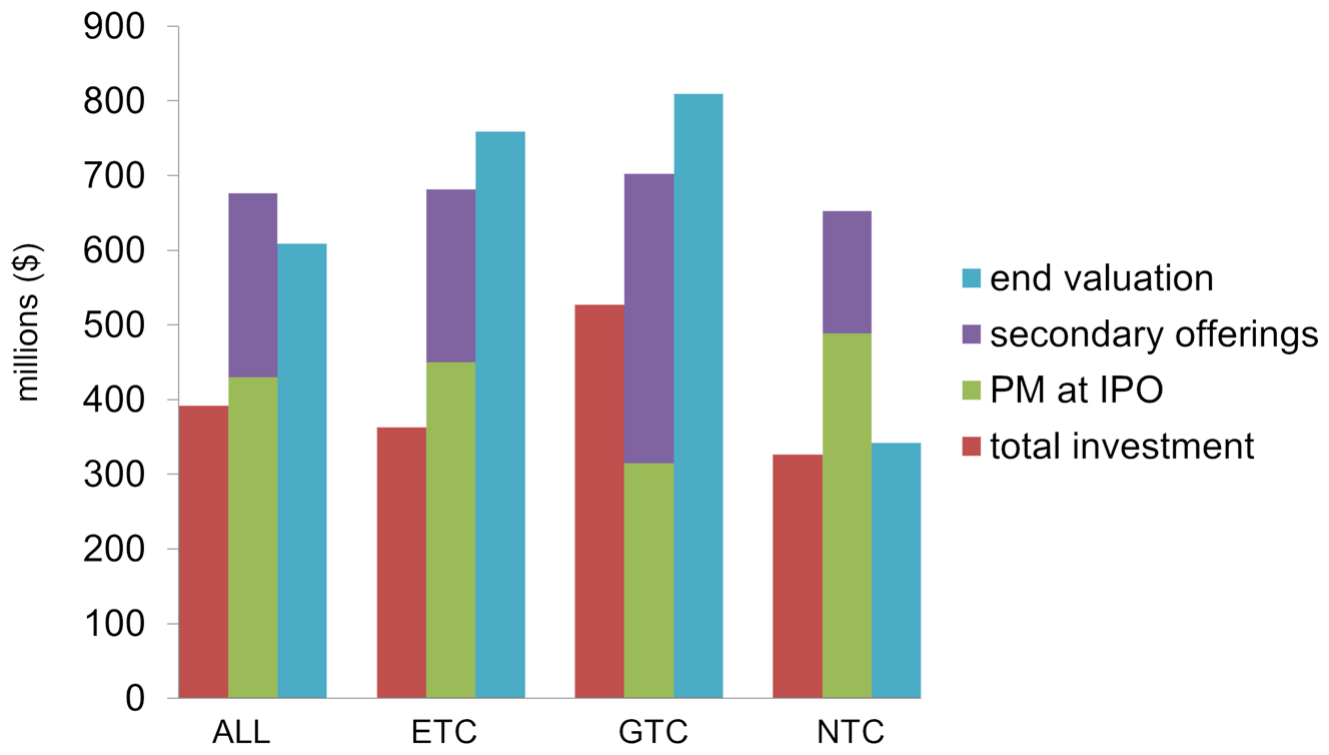
Capital investment and end valuations for each technology class. Average total investment (red bars); Average post-money (PM) valuation at IPO (green bars); Average secondary offering (purple bars); Average end valuation (blue bars). End Valuation of NTCs was significantly lower than those of GTCs, or ETCs and GTCs together (p<0.05).

**Figure 4:**
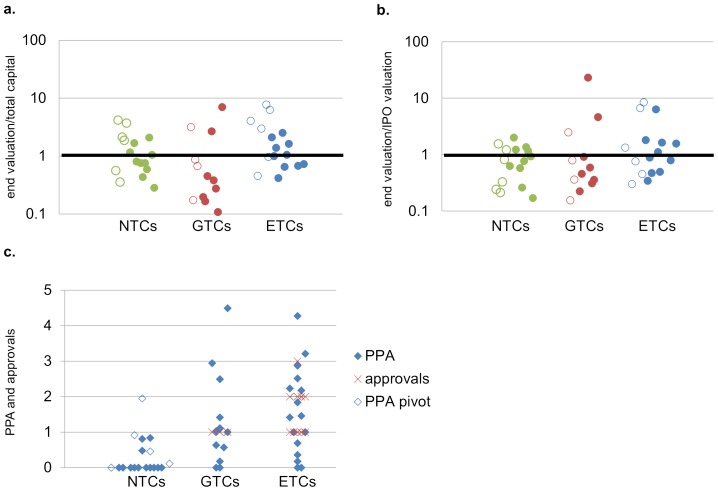
Snapshot of public value creation by the companies in the “class of 2000.” End Valuation of each company relative to (**A**) total capital investment and (**B**) post-money valuation at IPO for NTCs (green), GTCs (red), and ETCs (blue) (open circles represent acquired companies, filled circles represent non-acquired companies, two NTCs that declared bankruptcy not shown). (**C**) PPA and approvals for each company.

To assess capital growth, we calculated the ratio of End Valuation to the total capital investment and to the post-money valuation at IPO. Note that neither is a direct measure of shareholder value because they do not account for dilution from options, non-capital transactions, or acquisitions of spin-off companies. The End Valuation of the 46 companies was $28B (mean  = $609M), which is greater than the post-money valuation at IPO of $19B (mean  = $430M) and the $18B of capital they raised in total (mean  = $392M). Individually, 17 of 46 companies had End Valuations greater than their post-money valuation at IPO, and 21 had End Valuations greater than the total capital raised. There was a significant relationship between the maturity of a company's technology at IPO and its economic performance in the ensuing decade. GTCs had the highest average End Valuation (mean  = $810M), followed by ETCs (mean  = $759M) and NTCs with lowest average End Valuation (mean  = $342M) (Figure 3). The average End Valuation of NTCs was significantly lower than those of GTCs, or ETCs and GTCs together. The End Valuation for ETCs and GTCs averaged 1.9 times capital raised and 1.2 times their IPO valuation, while the average End Valuation for NTCs was equal to the capital raised, but only 0.7 times their IPO valuations. While the average return on investment was positive for GTCs relative to capital investment or their IPO valuation, the majority of GTCs had a net negative return on investment. A greater proportion of ETCs achieved positive End Valuations than either GTCs or NTCs. There was no average difference in the End Valuation between acquired and non-acquired companies (Figure 3, Figure 4A and B).

The positive return on investment achieved by GTCs and ETCs, as a group, is particularly notable during a difficult economic decade that saw the NBI drop from 1,619 in March 2000 to 901 in December 2010, and the NASDAQ composite drop from 5,132 to 2,653 over the same period. It is notable that, while the NTCs significantly underperformed companies with more mature technologies and provided a net negative return over the decade, they still outperformed both the NBI and NASDAQ over this period.

### Pivots and abandonment of *Nascent* technology

One of the striking observations in this cohort was that ten years after IPO, none of the 18 NTCs were still focused on advancing their core technologies. Five of these companies were focused on clinical development of products that had arisen from their core technology. Five others had abandoned their core technologies in favor of in-licensing late stage products or technologies, that would have been classified as *Growth* or *Established* in 2000. We term this a “Pivot.” Five other companies abandoned their *Nascent* technologies in conjunction with a merger or acquisition. Two companies that continued to focus on their core *Nascent* technologies declared bankruptcy. The remaining company was acquired by a large pharmaceutical company and retained its technology focus as an in-house research center.

### Product development

Of the 36 products described as being in clinical development in 2000 at the time of IPO, 9 were subsequently approved, 7 from ETCs and 2 from GTCs. Two additional products remain in phase 3 trials. This success rate correlates closely with the number of PPAs at the time of IPO (11) calculated from their stage of clinical development and the Probability of Success (POS) metrics reported by Tufts Center for the Study of Drug Development.[10] (Table 1).

Between 2000 and 2010, the companies in this study conducted clinical research on a total of 247 different entities, with the largest number by ETCs (110) and fewer by GTCs (83) or NTCs. At the end of 2010, a total of 17 products had been approved by the FDA, with ETCs launching significantly more products (14) than either GTCs (3) or NTCs (0) and having significantly more products in each phase of clinical development (Table 2). This total does not include entities that may have entered phase 1 after a company was acquired or approved products that were acquired subsequent to IPO.

**Table 2:** Products in clinical development at the end of the study.

	Phase 1	Phase 2	Phase 3	NDA	PPA	Approved
ETC	16	18	9	0	24	14
GTC	24	25	10	1	11	3
NTC	7	11	5	0	7	0

**Figure 5:**
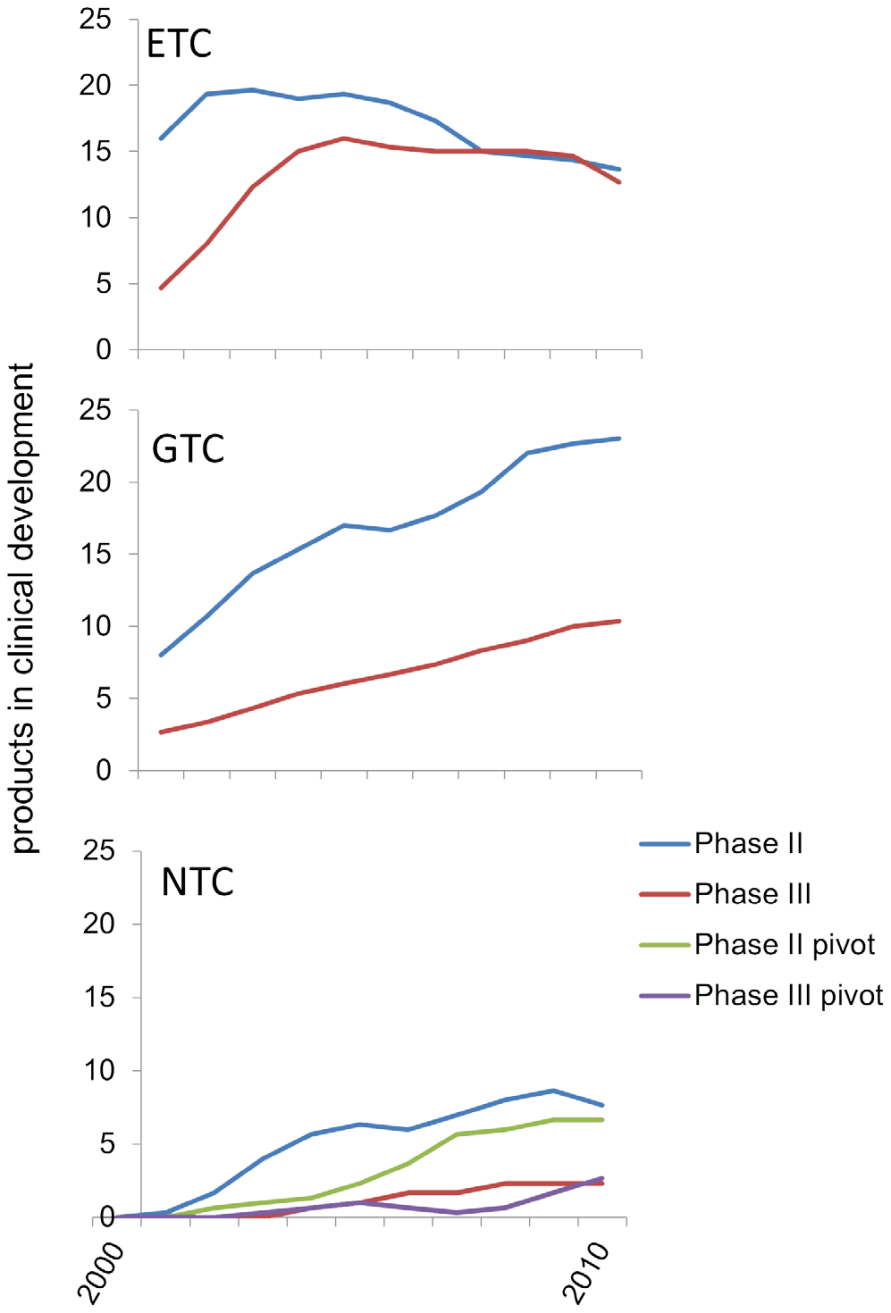
Average amount of products in active clinical development (Phase 2 or Phase 3). Three-year moving average for the number of products in Phase 2 or Phase 3 of clinical development for each technology cohort.

Figure 5 shows the number of products in phase 2 or phase 3 clinical development from IPO to the end of 2010. This figure shows that there is a progressive diminution of the ETC product pipelines as products are approved over time, while GTCs demonstrate steady growth of their clinical pipeline as well as the number of product approvals. NTCs achieved steady growth in the number of products in clinical trials, with many of these arising from a pivot to more mature technologies. Based on the number of products and their clinical stage in 2010, calculation of the PPA suggests that, in addition to the 17 products already approved, an additional 25 products would be launched from the 111 candidate products in clinical development; 10 by ETCs, 8 by GTCs, and 7 by NTCs. Therefore, the total PPA for all of the companies that completed IPOs in 2000 is 42 products, 24 by ETCs, 11 by GTCs, and 7 by NTCs (Table 2, Figure 4C). Significantly, the PPA of 7 for NTCs includes 4 from companies that were classified as a Pivot and do not derive products from technologies that were *Nascent* at the time of IPO. Thus, only three product approvals would be projected from the technologies that were *Nascent* in 2000.

## Discussion

This work asked whether the maturity of technologies through a quantifiable life cycle was a significant factor in product development and capital growth by public biotechnology companies, as it is in other technology-driven sectors. The biotech “class of 2000” provided an informative experimental model due to the fact that each of these companies was an established corporate entity, with management teams, capital resources, and technologies that were attractive to investment bankers and institutional investors, and were subject to the same market conditions, investment and partnering trends, and regulatory environment through the period studied.

These data suggest that while there is extensive variation in the performance of individual companies, there were statistically significant differences between the performance of companies whose technologies were *Nascent* at the time of IPO and those that had more mature *Growing* or *Established* stage technologies. Companies with *Nascent* technologies in 2000 had significantly poorer economic performance over the ensuing decade, generated an extremely limited product pipeline based on these technologies, and were less likely to generate capital growth. Thus, technological maturity through the technology life cycle correlates significantly with the ability of companies that completed an IPO in 2000 to successfully translate their core science and technology into products and capital growth.

The inability of these companies to launch products or generate capital growth from *Nascent* technologies is particularly striking given the nature of the study cohort, which comprised many of the highest profile biotechnology start-ups of the late 1990s. As many of the *Nascent* technologies were involved in genomics, we cannot rule out the possibility that these observations are specific to genomic technologies. The completion of the Human Genome Project in 2000 generated enormous optimism in the commercial opportunities enabled by genomics. As early as 2001, however, a report from Lehman Brothers raised concern that genes being discovered through genomics were not adequately validated, and that focusing on such targets would raise the cost of drug discovery and development in the near term.[11].

Recognizing that biotechnology is considered an inherently high risk and potentially high reward activity from an investment perspective, these results are particularly notable. Financial markets classically consider risk, which discounts the value of potential future markets, in calculating corporate valuations. The predicted impact of the risk associated with unproven, nascent technologies would be to suppress, rather than enhance, the valuation of companies at IPO. The present observations show that companies with nascent technologies were, in fact, significantly overvalued by investors relative to companies with more mature technologies. Presumably biotechnology investors with a choice of offerings therefore systematically underestimated the risk of companies with nascent technologies or overestimated their market opportunity, even though they had no products in clinical development and were furthest from having marketable products.

While 2000 was indeed a time of extraordinary investment activity, biotechnology companies have long been able to complete IPOs with nascent technologies. New waves of biotechnology, such as genetic engineering, the discovery of oncogenes, monoclonal antibodies, and rational drug design, are often heralded as having the potential to reduce the risk, cost, and time of drug development. Critical analysis, however, shows that each wave of new technology actually introduces increased product development and financial risk due to a lack of fully understanding that technology.[3], [11], [12] While current investment patterns may be more conservative than those seen in 2000, companies with nascent technologies such as RNA interference, epigenetics, cancer stem cells, and synthetic biology have continued to attract both public and private financing in the ensuing decade.

Previous research has emphasized the importance of a fit between the position of a technology in its life cycle and the business model designed to commercialize that technology. Chesbrough and Rosenbloom defined a business model as a “…coherent framework that takes technological characteristics and potentials as inputs, and converts them through customers and markets into economic outputs. The business model is thus…a focusing device that mediates between technology development and economic value creation.”[13] Their work highlighted the need for a fit between business models and technology in understanding why Xerox failed to commercialize many of the revolutionary technologies pioneered at its Palo Alto Research Center (PARC), while the same technologies were commercialized successfully by such companies as Apple, 3Com, and Adobe.[13] Similar concepts have been advanced by Christensen studying innovations in the steel, disc drive, and excavator industries.[6], [13].

While there are many components to a business model[14], the companies in this study each employed a model in which core technologies, private investments, and often alliances were leveraged to complete an IPO in the absence of commercial products or revenues. The present data suggests that models with these elements produced significantly different returns based on the maturity of the core technology. The companies in this cohort succeeded in creating economic growth from both *Established* and *Growth* stage technologies, though the proportion of failures was higher from *Growth* stage technologies. They also succeeded in generating a large number of new products from both *Established* and *Growth* stage technologies. In contrast, while companies with *Nascent* stage technologies achieved the highest step-up in valuation for venture investments at the time of IPO, they subsequently failed to generate either products or sustainable economic returns.

Evidence-based business models for the translation of nascent technologies should recognize that there is reproducible pattern of latency before new technologies lead to robust product pipelines and commercially viable products. We have previously observed that investments in monoclonal antibody technologies similarly failed to produce products or economic returns when these technologies were *Nascent*, and that product development began to be successful only as these technologies approached maturity.[8] A similar pattern is now emerging with regard to pharmacogenomics[15] and gene therapy.[16].

In this context, it is important to recognize that the “failure” of companies in this study to translate *Nascent* technologies into new products or economic value may reflect the inadequacy of the business model rather than failure of the technology itself. Pisano observed that biotechnology companies pursuing a “Science Business” may require business models with a distinctly different architecture and components than companies with more mature technology or companies in other sectors. He has proposed, for example, that the industry may need more “patient capital” to allow technologies to mature before committing to development.[3] For example, these data suggest that a more modest valuation of companies with *Nascent* technologies at IPO would have enabled these companies to provide returns indistinguishable from those of companies with more mature technologies. We would argue that the need for patience extends also to clinical development. For example, studies have shown that start-up companies are more likely than mature companies to move products into phase 2, but that these trials are more likely to fail[17].

While the cohort of companies used in this study provided a unique opportunity to examine how technological maturity impacts the translation of science into products and economic value, caution must be exercised in generalizing these findings. This is particularly true with respect to the IPO valuations in 2000, which may have been atypical. The present findings concerning product development and value creation by these companies between 2000 and 2010 (present time) may be more robust, since this period is most reflective of the contemporary biotechnology industry practices, investment and partnering trends, and regulatory environment. Further research will be required to examine the generalizability of these findings to other biotechnologies as well as changing market conditions, regulatory policies, and business practices. Of particular interest will be ten-year follow-up of the current (2012–2013) IPO window, which has seen more IPOs and a higher Biotechnology Index than in 2000.[18].

The biotechnology industry plays a central role in the translation of nascent biomedical science into both products that offer material health benefits to the public and create economic growth. The present results suggest that the maturity of a technology may be a critical determinant of the ability of companies to fulfill this role. Moreover, these results confirm other observations suggesting that the classical path for translational science, in which nascent advances arising from the scientific enterprise are transferred to early-stage companies that draw support from capital markets, is not effective in generating either new products or economic value. The increasing reluctance of investors to support early-stage start-ups, the emergence of incubators and translational medicine centers, and the commitment of the NIH to a National Center for Advancing Translational Science represent responses to the inefficiency of this older model. What is required is an evidence-based strategy for translational science that more effectively integrates technology and business to ensure efficient translation of scientific and technological advances to provide public benefit.

## Methods

### Company data

Biotechnology companies that completed an IPO between October 1999 and December 2000 (the “2000 IPO window”) and were involved in developing therapeutic or diagnostic products were identified in BioCentury Online Intelligence (BCIQ) database. This analysis excluded companies strictly focused on agriculture, instrumentation, or devices. A list of companies included in this study is shown in Table S1. Financial data for private and public financings were obtained from SEC filings and BCIQ. Market capitalization, operating income, and revenue post-IPO were retrieved from Bloomberg, Capital IQ, and SEC filings. Data on acquisitions and mergers were retrieved from BCIQ, SEC filings, or press releases. The NASDAQ Biotech Index was retrieved from http://www.google.com/finance. The Consumer Price Index (CPI) was retrieved from http://www.bls.gov/cpi/home.htm.

### Product development pipelines

Product development pipelines, clinical trial status, product approvals, and the terminal fates of products in development were obtained from PharmaProjects or SEC filings. Probability of Success (POS) values for the biopharmaceutical industry for the years 1993–1998 and 1999–2004 were obtained from Tufts Center for the Study of Drug Development [10]. The POS value is based on a set of products with a known terminal fate (either approval or discontinuation) and is calculated as a ratio of the number of products that enter a given phase to the number of products that move on to the subsequent phase. The Predicted Product Approval (PPA) was determined by multiplying the number of products in each stage of clinical development by the respective industry standard POS values for products at that phase [10] plus the number of products already approved.

### Technology life cycle metrics

Each company's core technology was characterized at the time of IPO based on descriptions in the S-1 filing and a rubric that considered both bibliometric analysis and clinical proof of concept. Bibliometric methods were based on the number of citations in the PUBMED database of the National Center for Biotechnology Information for the years 1960–2010. The cumulative number of publicationsover time was approximated by the log logistic regression equation. 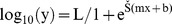
where y =  cumulative number of publications, L =  limit of log_10_(y), and x is years. The limit L is estimated as the value of L that gives the maximum R^2^ between the raw data y and the best fit logistic regression value Y*. First derivative and second derivative (d^2^Y*/dx^2^) were determined analytically.

A *Nascent* Technology is defined as one that is at or near the *Invention* stage of the technology life cycle approximated by the maximum of d^2^Y*/dx^2^and has not yet achieved clinical proof of concept. This corresponds generally to the metric of “first key paper” defined by Cockburn and Henderson[19]. A *Growing* Technology is one that is past the *Invention* stage of the technology life cycle in the exponential phase of the S-curve approximated by d^2^Y*/dx^2^ = 0 or one for which there is clinical proof of concept but no products approved or near approval. An *Established* Technology is one that is at or near the *Maturity* stage of the technology life cycle, approximated by the minimum of d^2^Y*/dx^2^, or one that has been incorporated in products that are approved or near approval.

### Statistical methods

Statistical methods included t-tests and chi-square. All calculations were done in MS Excel.

## Supporting Information

Table S1 A list of companies included in this study.
(TIF)
